# Markers of too little effort or too much alertness during neuropsychological assessment: Demonstration with perioperative changes

**DOI:** 10.1002/brb3.3649

**Published:** 2024-08-21

**Authors:** Dana Baron‐Shahaf, Goded Shahaf

**Affiliations:** ^1^ Neuro Anesthesia Unit Rambam Healthcare Campus Haifa Israel; ^2^ Applied Neurophysiology Laboratory Rambam Healthcare Campus Haifa Israel

**Keywords:** cognitive assessment, cognitive effort, EEG, monitoring, stress effect

## Abstract

**Objective:**

Cognitive assessment is based on performance in different tests. However, this performance might be hindered by lack of effective effort on the one hand, and by too much stress on the other hand. Despite their known impact, there are currently no effective tools for measuring cognitive effort or stress effect during cognitive assessment. We developed real‐time electrophysiological markers for cognitive effort and for stress effect, which could be used during cognitive assessment.

**Methods:**

We assessed these markers during the use of the Montreal Cognitive Assessment (MoCA) before and after cardiac surgery, which is known to involve cognitive decline in up to 30%–50% of elderly patients.

**Results:**

The major findings of the study, for the largest group of patients, with preoperative MoCA in the intermediate range, were that the decline is significantly associated (1) with higher preoperative cognitive effort and (2) with higher postoperative stress effect during the test.

**Conclusions:**

These findings, as well as preliminary additional ones, suggest a potential importance for monitoring cognitive effort and stress effect during assessment in general, and specifically during perioperative assessment.

**Significance:**

Easy‐to‐use markers could improve the efficacy of cognitive assessment and direct treatment generally, and specifically for perioperative decline.

## Graphical abstract

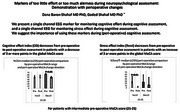

## 1 INTRODUCTION

Cognitive assessment is generally based on performance in different tests, which are designated to evaluate multiple cognitive functions. However, the performance in such tests might be hindered by lack of effective effort on the one hand (Boone, 2009), and by too much stress on the other hand (Dorenkamp et al., 2023).

Cognitive effort is often defined as the engagement of our limited cognitive resources in the task at hand (Tyler et al., 1979). However, despite an apparent intuitive grasp of the concept, there is significant variability in its operational definition (Westbrook & Braver, 2015). Still, even with the use of variable measurements of cognitive effort, its impact on performance in cognitive assessments has been well established, and too little cognitive effort may lead to underestimation of cognitive abilities (Hertel et al., 1979).

Cognitive effort might be affected by multiple clinical conditions. The ability to recruit it is lower in attention deficit hyperactivity disorder (ADHD; Pineda et al., 2007), and there appears to be significant avoidance from cognitive effort in depression (Vinckier et al., 2022) or under stress (Bogdanov et al., 2021). However, generally, and even in these and other clinical conditions, the emphasis in the literature is on motivation and volition, as the major underlying factors for cognitive effort (Nelson et al., 2007; Westbrook & Braver, 2015).

However, despite its significant impact on performance, it is often challenging to evaluate cognitive effort during cognitive assessments, and the evaluation of its impact upon the assessment results seems to be rather insufficient. Physiological measures have been offered for this aim, and mainly eye tracking of pupil size and of saccades, but their application during assessments might be cumbersome and limited (Krejtz et al., 2018). Electroencephalography (EEG) markers have also been suggested, but they often involve long samples, and evaluation of response to repetitive stimuli, which limit their use for many neuropsychological assessments (Umemoto et al., 2019).

In the opposite direction from reduced cognitive effort, too much stress might also hinder performance in cognitive assessment, which involves tasks that challenge the patient (Bierman et al., 2005; Needham et al., 2017). Stress might lead to enhanced cognitive effort, but an ineffective one, which is not allocated to the cognitive task, but potentially rather to the stressing factors (Ansari & Derakshan, 2011).

Stress too seems to be undermonitored during cognitive assessment, despite the fact that there are allegedly good physiological markers for stress, which measure the activation of the autonomic nervous system (Hickey et al., 2021). It seems that there is interindividual variability in the susceptibility of higher level cognitive performance to the impact of stress (Sandi, 2013). Therefore, it is not necessarily the autonomic stress response we may want to measure, but rather the impact of this stress upon the individual cognitive performance. For this aim too, some EEG‐based markers have been suggested (Angelidis et al., 2018), however, no such marker, for the impact of test‐related stress upon cognition, is thus far considered sufficiently convenient and effective for routine use during cognitive assessment.

Over the last few years, we have developed easy‐to‐use, single‐channel, and real‐time markers for evaluating cognitive effort and stress effect, which might be applicable also during cognitive assessment. We have developed markers for sustained attention, whose efficacy was demonstrated with multiple clinical populations and conditions (Baron Shahaf et al., 2022; Isserles et al., 2018; Shahaf et al., 2018, 2017). The efficacy of these markers was demonstrated by other research groups as well (Avirame et al., 2022; Bart & Liberman, 2020; Yogev‐Seligmann et al., 2022), and were also put to clinical use (Gvion & Shahaf, 2023; Karpin et al., 2022). A marker for the dynamics of sustained attention would be a good surrogate for cognitive effort during a cognitive task, as it is known that cognitive effort has a major impact on sustained attention (Massar et al., 2016; Shenhav et al., 2017). We currently use the cognitive effort index (CEI) for this aim (Baron Shahaf et al., 2022; Gvion & Shahaf, 2023). Our sustained attention markers emerged from the observation that transient changes in delta activity, over hundreds of milliseconds, might associate strongly with attention (Shahaf et al., 2015).

In the current study, we introduce, additionally, a novel marker for the evaluation of stress effect on cognitive performance—the tension index (TensI). This marker is based on real‐time changes in higher beta activity, which are measured in relation to a baseline level, extracted from periods of effective sustained attention. Palacios‐García et al. (2021) suggested that the higher beta activity, and especially the change of higher beta activity from baseline, might be related with the impact of stress upon attention and cognitive performance. We have accumulated experience with the TensI marker, in multiple samples from chronic pain patients and from patients with anxiety, which seem to be in agreement with their report. However, the current work is the first formal study to use the TensI marker.

The evaluation of the potential contribution of the CEI and of the TensI for the quality of neuropsychological evaluation was done by computing these indices during Montreal cognitive assessments (MoCA) before and after cardiac surgery. Cardiac surgery involves prevalent and measurable cognitive decline, or postoperative cognitive dysfunction/decline (POCD), especially in elderly patients, occurring in up to 30%–50% of the patients. MoCA is one of the tools that are being used to assess this decline, by comparing the test results before and after the surgery (Brodier & Cibelli, 2021). In this study, we evaluate whether and how such a decline might be associated with the perioperative measures of the CEI and of the TensI.

## 2 METHODS

### 2.1 Patients

A total of 120 patients who underwent elective cardiac surgeries in Rambam healthcare center, between January 2021 and December 2022, were included in this study, which was authorized by the institute review board (IRB). The study was a part of a larger study, involving also intraoperative monitoring, which was registered at clinicaltrials.gov (NCT04512989). All patients of any age who underwent elective cardiac surgery in Rambam during the study period, and who agreed to sign an informed consent, were eligible to participate in the study. For three patients, we did not have either the preoperative, or the postoperative data, so they were excluded from further analysis and the study sample included 117 patients. Due to some novelties, and particularly of one of the markers used, the sample size was not derived from a prestudy power analysis, but rather we sampled as many patients as we could during the study period.

### 2.2 Procedure

Patients underwent cognitive assessment twice, using the MoCA (Nasreddine et al., 2005), 1 day before the surgery, and during the first postoperative week, 1 day before they were discharged from hospitalization. The assessments were administered by three trained evaluators. For each patient, both preoperative and postoperative tests were administered by the same evaluator. Each test assessment was also reviewed by a trained physician.

During both the preoperative and postoperative MoCA tests, EEG sampling was performed with the Medtronic Bispectral Index (BIS) monitor, using a single‐channel sticker placed below the hairline at the left‐to‐middle forehead. After the test was complete, the raw EEG data were exported from the BIS monitor to a flash drive for offline analysis. The sampling was undertaken in a blinded manner without inspection of the BIS index, or of the continuous EEG waves, displayed by the BIS monitor.

Immediately prior to each cognitive assessment, the levels of the patient's anxiety and depression were evaluated, using the Hospital Anxiety and Depression Scale (HADS) questionnaire (Zigmond & Snaith, 1983). The EEG recording start time, and the relative start and end times of the active MoCA test were documented for each sample, and were used to delimit the MoCA sample for analysis.

### 2.3 Data analysis

#### 2.3.1 MoCA: Ranges and subscores

The precise division of MoCA scores to ranges of normal, mild cognitive impairment (MCI) and dementia are still being reviewed (Carson et al., 2018). Based on this long standing literature, it seems reasonable to conclude that scores of 26 or above are in high likelihood normal, and scores of 20 or below are in all likelihood abnormal, and patients in this lower range suffer from dementia or at least from MCI (Dautzenberg et al., 2020). Therefore, in order to assess the impact of preoperative cognitive status upon the results, we divided a priori the patients into three preoperative MoCA ranges: lower—MoCA ≤ 20, intermediate—21 ≤ MoCA ≤ 25, and higher—26 ≤ MoCA.

#### 2.3.2 Computation of the CEI

The EEG sample during MoCA was divided into 10 s segments. Each 10‐s segment was filtered to the delta band (1–4 Hz), and then the filtered segment was divided into 20 epochs of 500 ms each. For each epoch, we computed the power of delta activity (based on integration of the absolute raw data—see also Baron Shahaf et al., 2022, fig. 1), and then we computed the mean and standard deviation of all epochs within a segment. Then the CEI was derived from the standard deviation to mean ratio and is in the [0,1] range. We learned that if the ratio is >1, it is likely to be due to a noisy sample, in which case no value was returned for this 10‐s segment (for more details regarding the CEI computation please see the relevant methods section in Baron Shahaf et al., 2022).

For each sample from a single MoCA test, we computed a global %CEIm value, representing the percent of CEI points in the middle third (between 1/3 and 2/3) out of the entire CEI test sample. Our experience with the CEI indicates that values in this range represent an effective attentional effort, while values below 1/3 may represent too low effort, and values above 2/3 may represent attention to a stressing stimulus (see more details in Gvion & Shahaf, 2023).

#### 2.3.3 Computation of TensI

For each 10‐s segment, we also computed the power of high beta band activity (23–30 Hz). Segments in which the CEI value (see above) was in the middle third (between 1/3 and 2/3) were considered as baseline segments, and the mean baseline beta power from these segments was updated with every additional baseline segment during the MoCA period. TensI for a given segment was derived from the ratio between the power of beta activity in the given segment and the mean beta power of the baseline segments preceding it. The TensI value was this ratio divided by two—so that when the current segment beta power was equal to the mean baseline beta power, TensI was 0.5. If TensI was greater than 1, it was set as 1. Thus, TensI was limited to the [0,1] range. The specification of baseline activity, according to segments with CEI in the middle, or effective attentional effort range follows our pilot experience with multiple clinical patients, and seems to accord with the experience of other researchers as well (Palacios‐García et al., 2021).

For each sample from a single MoCA test, we computed a global %TensI↑ (read as %TensI‐high) value, representing the percent of TensI points in the higher third (greater than 2/3) out of the entire TensI test sample. Based on our experience and the rationale, which was presented by Palacios‐García et al. (2021), %TensI↑ may represent the degree of enhanced alertness, or possibly of anxiety during the measured MoCA test.

The approach of excluding segments, in which the standard deviation was greater than the mean, reduces overt noise due to intense muscle activity (Baron Shahaf et al., 2022). However, especially when prefrontal recording is considered, there is always concern regarding the impact of “milder” Electomyography (EMG) noise sources. There seems to be a range of overlap in which it is uncertain whether activity originates from the brain or from muscle activity. Specifically, for the CEI, and the delta activity in this region, there is a major concern regarding the effect of blinking (Delorme et al., 2007). However, interestingly, blinking is well related to attention (Maffei & Angrilli, 2018). Furthermore, it seems the pattern of “attentive” well‐deferred blinking in the delta band pass may lead to greater variability of the signal, which would also be captured by the CEI marker (Baron Shahaf et al., 2022). Therefore, we did not see a practical need to differentiate between EEG activity and blinking. For the TensI, and the high beta activity, in this region, there is a major concern regarding the effect of the frontalis muscle contraction (Goncharova et al., 2003). However, interestingly, increased frontalis contraction is related to stress (Orr et al., 1993), which would be captured by the TensI marker. Therefore, we did not see also a practical need to differentiate between EEG activity and frontalis contraction.

### 2.4 Statistical analysis

Following early computations, both %CEIm and %TensI↑ do not demonstrate normal distribution. Therefore, we utilized the Mann–Whitney test for the assessment of significant differences in these two electrophysiological markers, as they were measured during pairs of MoCA tests, such as preoperative tests versus postoperative tests, or tests of patients who showed MoCA decrease versus tests of patients who showed MoCA increase.

For the assessment of differences in patient age, in HADS score, and in MoCA subscores, we used *t*‐test, due to the accepted normal distribution of these variables.

For the assessment of the differentiation between patients with perioperative MoCA decrease and patients with perioperative MoCA increase on the basis of %CEIm and %TensI↑ thresholds, we used the chi‐square test.

## 3 RESULTS

Table 1 presents the counts of patients who showed a decrease or an increase in the MoCA score between the preoperative assessment and the postoperative assessment. The counts are presented for each category range of the preoperative MoCA score assessment: MoCA ≤ 20, 21 ≤ MoCA ≤ 25, and 26 ≤ MoCA. For each range, the table presents the number of patients with MoCA decrease or increase with thresholds of either one, two, or three points. All patients with at least a three points change are also included in the group of patients with at least a two points change, and all these patients are also included in the group of patients with at least a one point change. It is noteworthy that only about 9% of the patients, or 11 patients altogether, did not demonstrate a perioperative MoCA change. Since the definition of POCD in terms of change in cognitive score is under debate and review (Rudolph et al., 2010), and since we do not want to undertake assumptions in this regard in the current manuscript, we simply excluded these 11 patients from further analysis. For the remaining 106 patients we then compared between patients with MoCA decrease of at least one point, of at least two points, and of at least three points, and patients with MoCA increase of the same thresholds, respectively. Beyond the three points threshold, the counts seem too small to support comparison. The results reported below involve the remaining 106 patients.

**TABLE 1:** Number of patients with different thresholds of perioperative Montreal Cognitive Assessment (MoCA) score change.

Preoperative MoCA range	Decrease ≥ three points	Decrease ≥ two points	Decrease ≥ one point	No change	Increase ≥ one point	Increase ≥ two points	Increase ≥ three points
≤20	4	9	13	4	15	8	6
21–25	9	15	20	4	44	27	13
≥26	2	4	8	3	6	4	1
Total	15	28	41	11	65	39	20
Percent of all	13%	24%	35%	9%	56%	33%	17%

Thresholds range from three or more MoCA point perioperative change, of either increase or decrease, to one (any) MoCA point change. The number of patients without perioperative MoCA change are presented in gray in the middle column. The patient counts are presented for each preoperative MoCA range—MoCA ≤ 20, 21 ≤ MoCA ≤ 25, and 26 ≤ MoCA.

Table 2 compares the demographic and affective data of the patients who showed a decrease and the patients who showed an increase, of at least one point, over the different preoperative MoCA ranges. The variables, which were compared between these groups are age, gender, preoperative HADS scores (total, anxiety, and depression), and postoperative HADS scores. Statistical significance was assessed with *t*‐test. The vast majority of comparisons were not significant. One significant difference was found between patients with MoCA decrease and patients with MoCA increase, in the group with preoperative MoCA score in the higher range (≥26). Among these patients, the ones who demonstrated a decrease in MoCA score were significantly older than the patients who demonstrated an increase in MoCA score. Another significant difference was in the postoperative HADS depression score, in the group with preoperative MoCA score in the lower range (≤20). The patients in this group, who demonstrated a decrease in MoCA score, reported significantly more postoperative depressive symptoms than the patients who demonstrated an increase in MoCA score. However, no other significant differences in affective symptoms, either of depression or of anxiety, were found between patients with MoCA decrease and patients with MoCA increase. It seems reasonable to conclude that no comprehensive association was found between general affective status, either before or after surgery, and the direction of MoCA change.

**TABLE 2:** Demographic and affective comparison of patients with Montreal Cognitive Assessment (MoCA) decrease versus increase.

				Pre			Post		
Preoperative MoCA range	Decrease or increase	Age (years) Mean ± SD	Male:female	HADS total Mean ± SD	HADS anxiety Mean ± SD	HADS depression Mean ± SD	HADS total Mean ± SD	HADS anxiety Mean ± SD	HADS depression Mean ± SD
≤20	Decrease	68 ± 8	10:3	14.8 ± 8.7	8.2 ± 6.2	6.7 ± 3.6	17.8 ± 12.0	8.1 ± 6.0	**9.8 ± 6.4**
≤20	Increase	66 ± 7	10:4	14.1 ± 5.9	8.0 ± 3.9	6.1 ± 3.2	12.9 ± 7.0	7.9 ± 4.9	4.9 ± 3.6*
21–25	Decrease	65 ± 10	14:6	10.5 ± 8.4	6.4 ± 4.8	4.1 ± 4.5	13.9 ± 9.0	7.1 ± 4.3	6.8 ± 5.5
21–25	Increase	63 ± 11	35:10	11.1 ± 6.5	6.1 ± 3.7	5.0 ± 4.1	11.1 ± 8.0	5.2 ± 4.3	5.8 ± 4.7
≥26	Decrease	**69 ± 9**	7:1	6.3 ± 4.7	4.6 ± 3.5	1.6 ± 1.7	10.3 ± 5.7	6.0 ± 3.0	4.3 ± 3.5
≥26	Increase	50 ± 14*	6:0	7.2 ± 3.8	4.5 ± 2.1	2.7 ± 2.0	11.5 ± 6.3	6.2 ± 3.7	5.3 ± 3.0

Comparison of age, gender, preoperative Hospital Anxiety and Depression Scale (HADS) scores, and postoperative HADS scores between patients with perioperative MoCA decrease and patients with perioperative MoCA increase. Patients with any decrease or increase (of one point or more) were included. The comparison was done for each preoperative MoCA range. Significant differences are marked in gray.

^*^ *p* < .05.

Table 3 compares the preoperative and the postoperative MoCA subscores. MoCA includes seven subscores: executive–visuospatial, naming, attention, language, abstract, memory, and orientation (Julayanont & Nasreddine, 2017). The subscores were compared for each of the preoperative MoCA ranges, for patients with an overall MoCA decrease (upper table) and for patients with an overall MoCA increase (lower table). Statistical significance was assessed with a paired *t*‐test.

**TABLE 3:** Perioperative changes in Montreal Cognitive Assessment (MoCA) subscores in patients with overall MoCA decrease or increase.

Decrease	Executive/visuospatial		Naming		Attention		Language		Abstract		Memory		Orientation	
Preoperative MoCA range	Pre Mean ± SD	Post Mean ± SD	Pre Mean ± SD	Post Mean ± SD	Pre Mean ± SD	Post Mean ± SD	Pre Mean ± SD	Post Mean ± SD	Pre Mean ± SD	Post Mean ± SD	Pre Mean ± SD	Post Mean ± SD	Pre mean ± SD	Post Mean ± SD
≤20	2.4 ± 0.9	2.1 ± 1.4	2.7 ± 0.7	2.7 ± 0.7	**4.5 ± 1.1**	**3.6 ± 1.1** *	0.6 ± 0.8	0.3 ± 0.5	0.8 ± 0.7	0.6 ± 0.7	0.8 ± 0.8	1.1 ± 1.5	**5.8 ± 0.5**	**4.5 ± 1.0** ***
21–25	**3.4 ± 1.0**	2.5 ± 1.1 ***	3.0 ± 0.2	2.9 ± 0.4	**5.7 ± 1.0**	**4.5 ± 1.3** ***	1.4 ± 0.9	1.1 ± 1.0	1.4 ± 0.8	1.5 ± 0.7	2.7 ± 1.3	2.7 ± 1.4	**6.0 ± 0.7**	**5.3 ± 0.8** **
≥26	**4.5 ± 0.8**	**3.9 ± 0.8** *	3.0 ± 0.0	3.0 ± 0.0	6.0 ± 0.0	5.9 ± 0.4	**2.1 ± 0.8**	**1.3 ± 0.9** *	1.9 ± 0.4	1.9 ± 0.4	3.9 ± 1.2	3.6 ± 1.2	6.0 ± 0.0	5.8 ± 0.5

Increase	Executive/visuospatial		Naming		Attention		Language		Abstract		Memory		Orientation	
Preoperative MoCA range	Pre Mean ± SD	Post Mean ± SD	Pre Mean ± SD	Post Mean ± SD	Pre Mean ± SD	Post Mean ± SD	Pre Mean ± SD	Post Mean ± SD	Pre Mean ± SD	Post Mean ± SD	Pre Mean ± SD	Post Mean ± SD	Pre Mean ± SD	Post Mean ± SD
≤20	2.8 ± 1.1	2.5 ± 1.1	2.8 ± 0.4	2.9 ± 0.4	**3.9 ± 1.1**	4.7 ± 0.8*	0.4 ± 0.6	0.4 ± 0.8	0.9 ± 0.8	1.0 ± 0.7	**0.8 ± 1.1**	2.2 ± 1.6**	5.4 ± 1.9	5.5 ± 0.7
21–25	3.6 ± 1.0	3.7 ± 1.0	3.0 ± 0.2	3.0 ± 0.3	5.4 ± 0.6	5.4 ± 0.8	1.6 ± 0.9	1.7 ± 0.9	1.5 ± 0.6	1.6 ± 0.6	**1.9 ± 1.5**	3.7 ± 1.0****	5.9 ± 0.3	5.8 ± 0.5
≥26	4.3 ± 0.8	4.7 ± 0.5	3.0 ± 0.0	3.0 ± 0.0	6.0 ± 0.0	6.0 ± 0.0	2.2 ± 1.0	2.3 ± 0.8	2.0 ± 0.0	2.0 ± 0.0	**3.3 ± 1.4**	4.8 ± 0.4**	6.0 ± 0.0	5.8 ± 0.4

Patients with any decrease or increase were included. The comparison was done for each preoperative MoCA range. The upper table is for overall perioperative MoCA decreases and the lower table is for overall perioperative MoCA increases. Significant differences are marked in gray, and level of significance in coded with asterisks.

^*^ *p* < 0.05; ^**^ *p* < .01; ^***^ *p* < .001; ^****^ *p* < .0001.

With regard to overall MoCA decrease, no single subscore showed a significant decrease for all three preoperative MoCA ranges. The orientation subscore decreased most significantly in patients with lower range preoperative MoCA, and also significantly in patients with intermediate range preoperative MoCA. The attention subscore decreased most significantly in patients with intermediate range preoperative MoCA, and also significantly in patients with lower range preoperative MoCA. The executive–visuospatial subscore decreased most significantly in patients with intermediate range preoperative MoCA, and also significantly in patients with higher range preoperative MoCA. The language subscore decreased significantly in patients with higher range preoperative MoCA. Ordering the significant subscore decreases by baseline (preoperative) MoCA range might indicate a rank in level of difficulty of the subscore tasks, where orientation tasks might be the easiest, and therefore are the most sensitive to decrease in the lower preoperative range, followed by the attention tasks, and then by the executive–visuospatial tasks, and the language tasks might be the most difficult. There was not a single subscore, which dominated the decrease across preoperative MoCA ranges, and which may indicate a highly specific neuropsychological profile for POCD.

With regard to overall MoCA increase, the situation seems quite different, and the most robust finding seems to be that the memory subscore showed a significant increase for all three preoperative MoCA ranges, possibly indicating learning between test and retest as a major mechanism of MoCA increase. There was only one other subscore with a significant increase—the attention subscore for patients with lower range preoperative MoCA. Interestingly, as was presented above (Table 2), this group of patients was the only one who demonstrated significantly less postoperative depressive symptoms than their counterparts with overall MoCA decrease.

**FIGURE 1:**
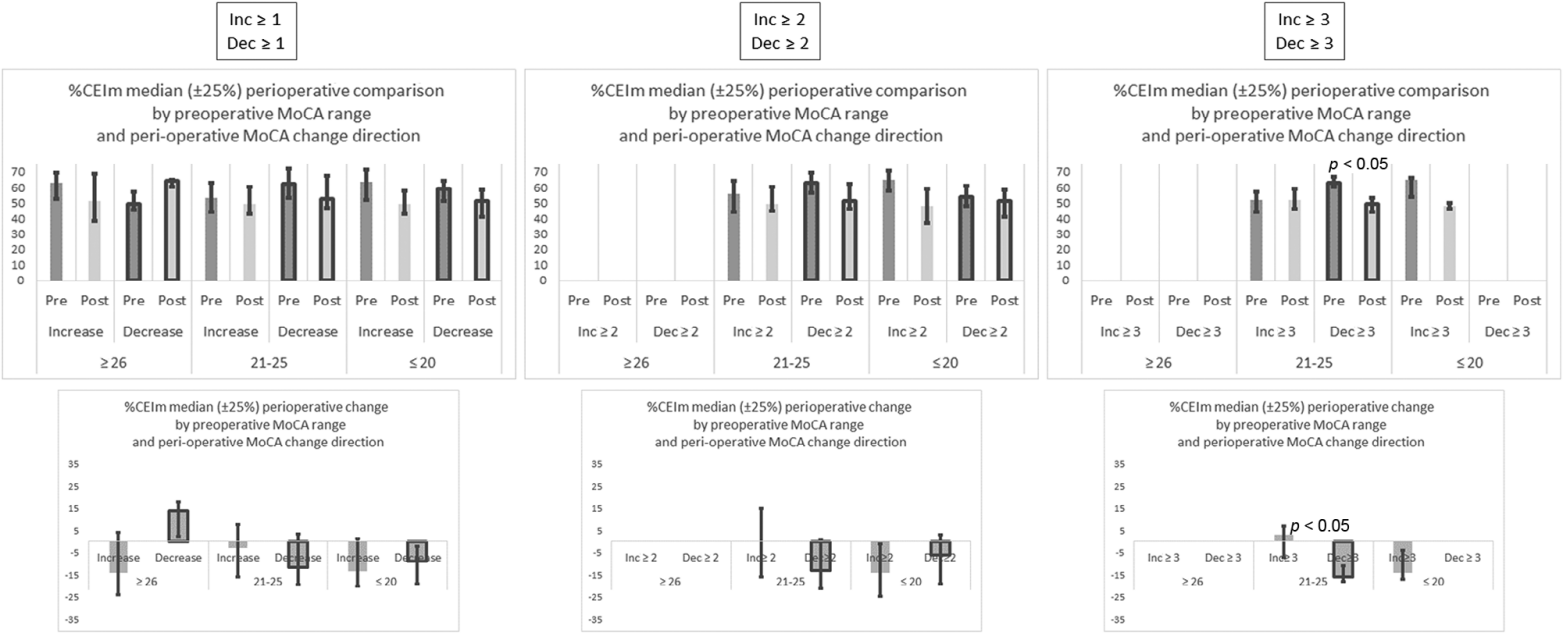
Comparison between preoperative and postoperative values of %CEIm during Montreal Cognitive Assessment (MoCA). The comparison is for three thresholds of perioperative difference in MoCA score. The left column is for any difference (≥1), the middle column is for differences ≥2, and the right column is for differences ≥3. Both graphs in each column are divided according to preoperative MoCA range, and then to increases and decreases in perioperative MoCA score. The upper graph in each column presents the preoperative %CEIm and postoperative %CEIm, and the lower graph in each column presents the **intrapatient** perioperative %CEIm change between the preoperative sample and the postoperative sample. For all values the median and the interquartile interval are presented. Only bars with at least five samples are presented, and therefore some bars are missing in the graphs of higher perioperative MoCA difference thresholds. The *p* values of significant differences are presented in the graphs.

Figure 1 presents the comparison of the perioperative change in %CEIm for patients with MoCA increase versus patients with MoCA decrease, at the three different levels of change: of at least one point, of at least two points, and of at least three points. In each case, the comparison is presented for the three preoperative MoCA ranges—lower (MoCA ≤ 20), intermediate (21 ≤ MoCA ≤ 25), and higher (26 ≤ MoCA). The upper graphs of the figure present the preoperative and postoperative medians and interquartile intervals of %CEIm values, and the lower graphs present the medians and interquartile interval values of the intrapatient deltas between the postoperative %CEIm and the preoperative %CEIm. Statistical significance was assessed with the Mann–Whitney test. When the change was of at least three points, the intermediate range patients with MoCA decrease demonstrated a significant reduction in %CEIm (upper graph), and also differed significantly from the intrapatient %CEIm delta in patients with MoCA increase (lower graph).

**FIGURE 2:**
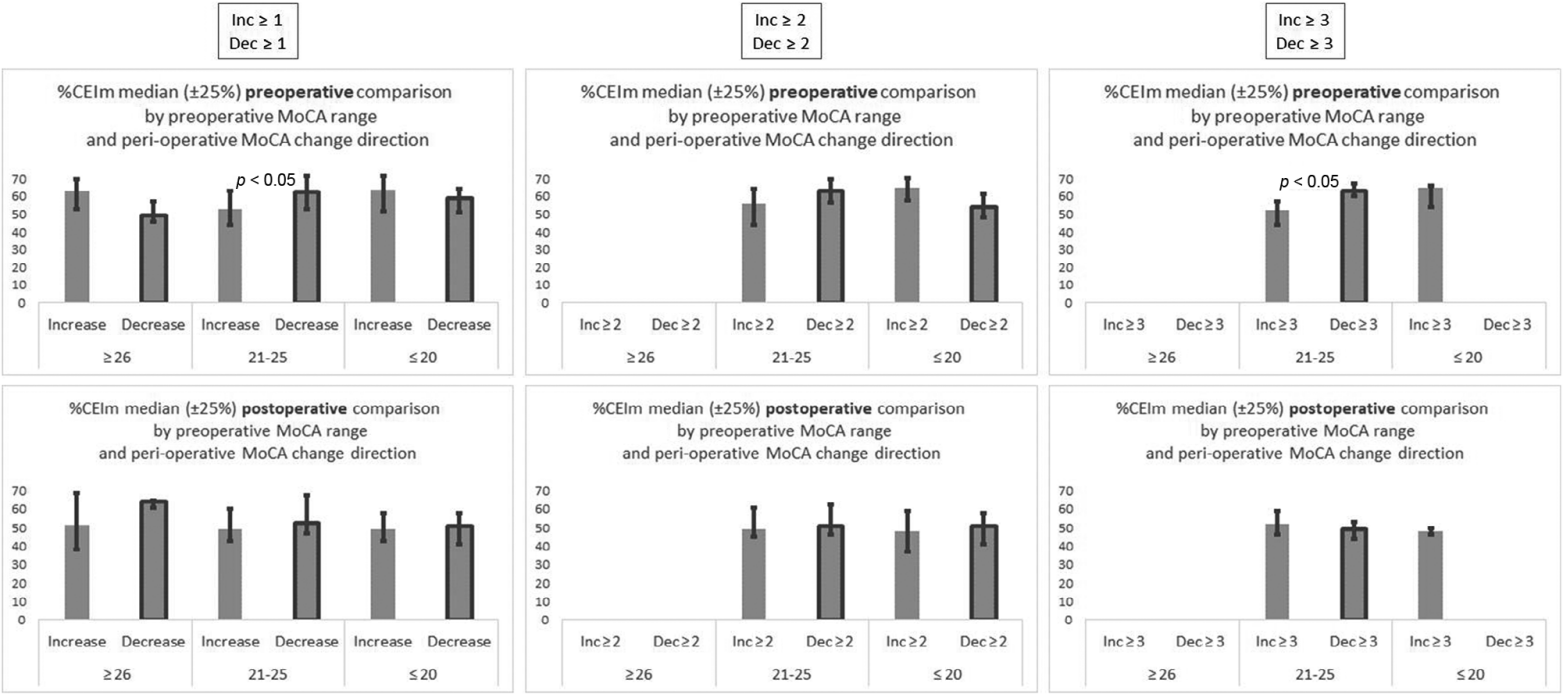
Comparison between increases and decreases of pre‐ and postoperative %CEIm values. The comparison is for different thresholds of perioperative differences in Montreal Cognitive Assessment (MoCA) score. The left column is for any difference (≥1), the middle column is for differences ≥2, and the right column is for differences ≥3. Both graphs in each column are divided according to preoperative MoCA range. The upper graph in each column presents the **preoperative** %CEIm for samples, which showed perioperative increase, and for samples, which showed perioperative decrease in MoCA score. The lower graph in each column presents the **postoperative** %CEIm for samples, which showed perioperative increase, and for samples, which showed perioperative decrease in MoCA score. For all values, the median and the interquartile interval are presented. Only bars with at least five samples are presented, and therefore some bars are missing in the graphs of higher perioperative MoCA differences. The *p* values of significant differences are presented in the graphs.

In Figure 2, it may be seen that this significant reduction in %CEIm could be associated with a significantly higher preoperative %CEIm in this specific group of patients. The figure presents the comparison of the preoperative (upper graphs) and postoperative (lower graphs) differences of %CEIm values in patients with the three levels of perioperative MoCA change, comparing patients with MoCA increase to patients with MoCA decrease. Statistical significance was assessed with the Mann–Whitney test. Significant differences in %CEIm were found between intermediate range MoCA increases and decreases in the preoperative sample, but not in the postoperative sample. The significant difference was found when comparing at least three point MoCA increases and decreases, but also when comparing any increases and decreases (at least one point).

With regard to the other preoperative MoCA ranges, it is interesting to note that while the differences did not reach statistical significance, possibly due to a smaller sample size, there might have been an opposite tendency, from the one found in the intermediate preoperative MoCA range. For the lower range, it might be possible to note a tendency of a higher preoperative %CEIm, in the preoperative MoCA test, in patients who showed an increase in their MoCA score, when compared to those who showed a MoCA decrease, with a threshold of at least two points. For the higher range, there may have also been a tendency of a higher preoperative %CEIm in patients who showed an increase in their MoCA score, compared to patients with MoCA decrease, with a threshold of at least one point. This one‐point threshold was the only one with enough (≥5) samples of both patients with MoCA increase and patients with MoCA decrease for this preoperative MoCA range.

**FIGURE 3:**
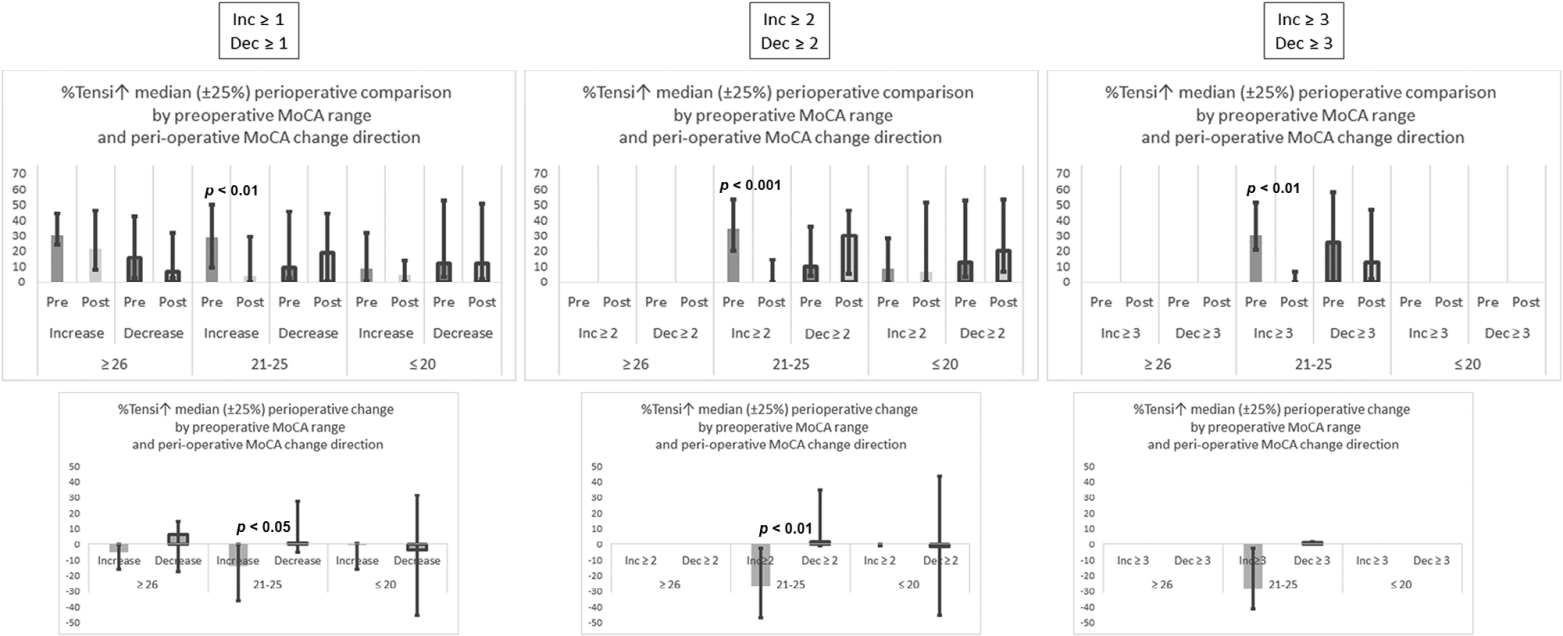
Comparison between preoperative and postoperative values of %TensI↑ during Montreal Cognitive Assessment (MoCA). The comparison is for different thresholds of perioperative difference in MoCA score. The left column is for any difference (≥1), the middle column is for differences ≥2, and the right column is for differences ≥3. Both graphs in each column are divided according to preoperative MoCA range, and then to increases and decreases in perioperative MoCA score. The upper graph in each column presents the preoperative %TensI↑ and postoperative %TensI↑, and the lower graph in each column presents the **intrapatient** perioperative %TensI↑ change between the preoperative sample and the postoperative sample. For all values, the median and the interquartile interval are presented. Only bars with at least five samples are presented, and therefore some bars are missing in the graphs of higher perioperative MoCA differences. The *p* values of significant differences are presented in the graphs.

Figure 3 presents the comparison of the perioperative change in %TensI↑ for patients with MoCA increase versus patients with MoCA decrease for the three different levels of MoCA change, of at least one, two, and three points. In each case, the comparison is presented for the three preoperative MoCA ranges. The upper graphs in the figure present the preoperative and postoperative medians and interquartile interval values, and the lower graphs present the medians and interquartile interval values of the intrapatient deltas between the postoperative %TensI↑ and the preoperative %TensI↑. Statistical significance was assessed with the Mann–Whitney test. Patients in the intermediate range of the preoperative MoCA, with MoCA increase, demonstrated a significant perioperative reduction in %TensI↑ for all levels of perioperative MoCA change. As could be seen in the lower graphs, this %TensI↑ reduction was significantly different from the perioperative %TensI↑ change in the patients with MoCA decrease, for perioperative MoCA changes of at least one point, or of at least two points, but the difference was not significant for a perioperative MoCA change of at least three points, possibly due to smaller sample size.

In the lower graphs, there might also be perioperative reductions in %TensI↑ in the higher and lower preoperative MoCA ranges, but these possible reductions seems to be more humble, and do not reach statistical significance for either of these ranges.

**FIGURE 4:**
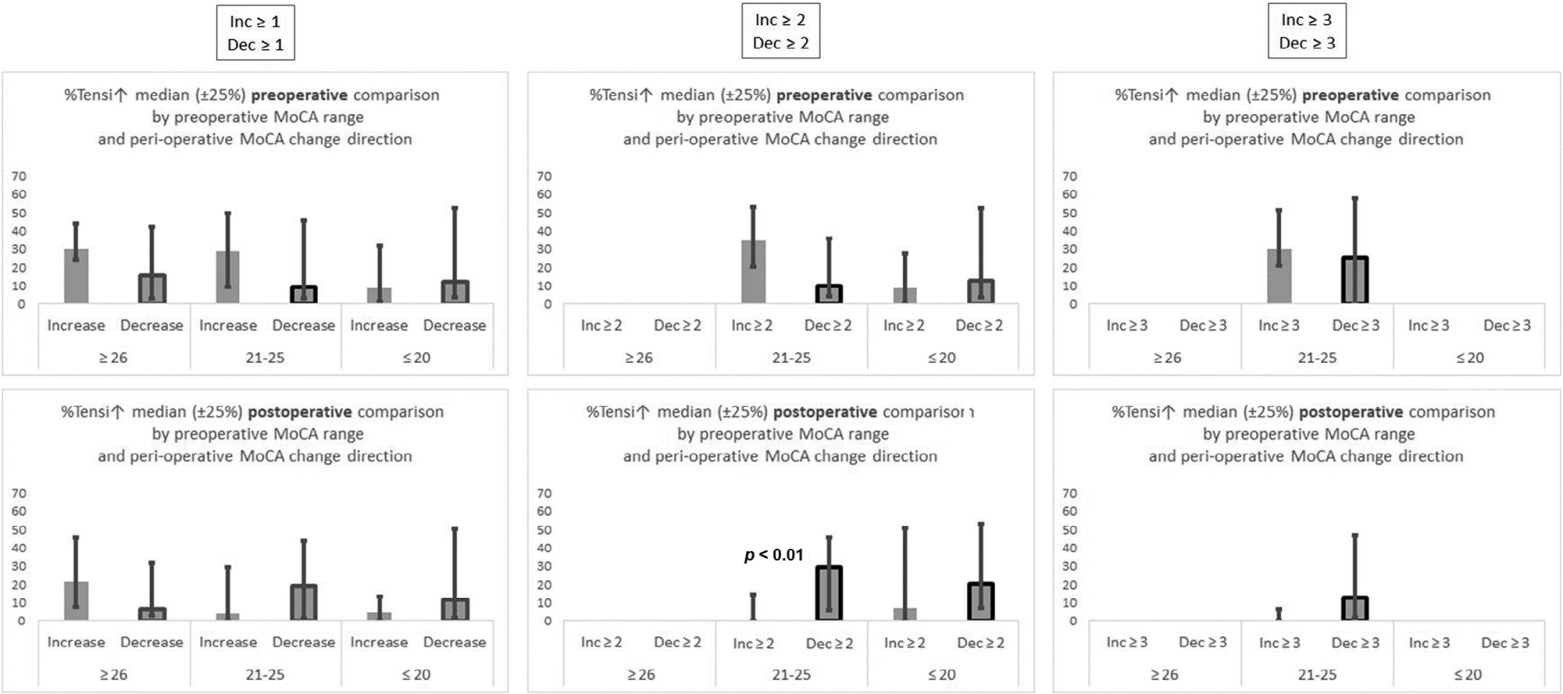
Comparison between increases and decreases of pre‐ and postoperative %TensI↑ values. The comparison is for different thresholds of perioperative differences in Montreal Cognitive Assessment (MoCA) score. The left column is for any difference (≥1), the middle column is for differences ≥2, and the right column is for differences ≥ 3. Both graphs in each column are divided according to preoperative MoCA range. The upper graph in each column presents the **preoperative** %TensI↑ for samples, which showed perioperative increase, and for samples, which showed perioperative decrease in MoCA score. The lower graph in each column presents the **postoperative** %TensI↑ for samples, which showed perioperative increase, and for samples, which showed perioperative decrease in MoCA score. For all values, the median and the interquartile interval are presented. Only bars with at least five samples are presented, and therefore some bars are missing in the graphs of higher perioperative MoCA differences. The *p* values of significant differences are presented in the graphs.

Figure 4 shows there is a significantly higher postoperative %TensI↑ in patients with MoCA decrease. The figure presents the comparison of the preoperative (upper graphs) and postoperative (lower graphs) differences between %TensI↑ values of patients with the three levels of MoCA increase in comparison to patients with MoCA decrease. Statistical significance was assessed with the Mann–Whitney test. A significantly higher postoperative %TensI↑ was found for MoCA decreases of at least two points, when compared with MoCA increases. Notably, there was no significant difference for a perioperative MoCA change of at least three points, and it seems that the median the postoperative %TensI↑ in the decreases group might be smaller than in the two points threshold. However, the sample size with the three‐point threshold seems too small for reaching statistical conclusions.

There might be a similar tendency toward higher postoperative %TensI↑ in decreases also for the lower preoperative MoCA range, but not for the higher range. However, the difference between the decreases group and the increases group does not reach statistical significance in both of these ranges.

**FIGURE 5:**
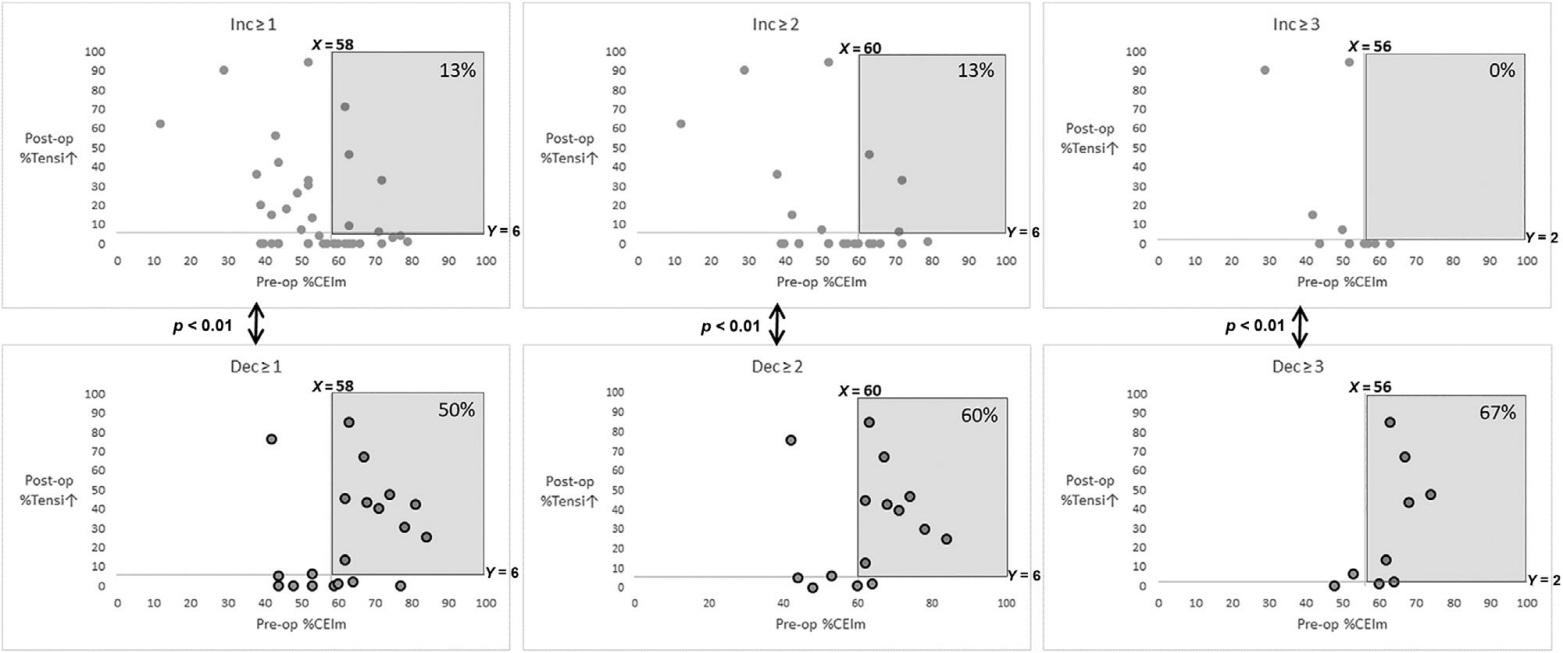
Comparison between increases and decreases by pre‐op %CEIm X post‐op %TensI↑ of patients with pre‐op Montreal Cognitive Assessment (MoCA) in the 21–25 range. The comparison is for different thresholds of perioperative difference in MoCA score. The left column is for any difference (≥1), the middle column is for differences ≥2, and the right column is for differences ≥3. In each column, the upper graph presents the patients with MoCA increase, and the lower graph presents the patients with MoCA decrease. Each patient's data are captured by a point in the graph. The *x* value of the patient's point denotes the %CEIm score and the *y* value denotes the %TensI↑ score. The two graphs of each column are divided into the same quartiles. The horizontal division is by the median %CEIm value of all patients in this column (with both MoCA increase and MoCA decrease), and the vertical division is by the median %TensI↑ value of all patients in this column. Thus, the divisions are identical for the two graphs in each column. The dividing medians are presented for each graph by *x* = for the median of %CEIm and *y* = for the median of %TensI↑. In each graph, the quarter marked in gray denotes the patients with both %CEIm and %TensI↑ above these median thresholds. The percent of patients in this quarter, out of the total patients in the group, is presented. The *p* value between the graphs in each column indicates the statistical significance of the difference in the ratio of patients in the gray quarter with MoCA decrease and patients with MoCA increase.

In the comparisons above, it was found that in the intermediate preoperative MoCA range there were one significant preoperative difference and one significant postoperative difference between patients with MoCA decrease and patients with MoCA increase. In patients with MoCA decrease, the preoperative %CEIm and the postoperative %TensI↑ were significantly higher. Figure 5 presents the interaction of these two indices. Each graph presents the two indices of all patients in the intermediate preoperative MoCA range—the *x* value of each patient point denotes the patient's %CEIm value, and the *y* value denotes patient's %TensI↑. The upper graphs present the data of patients with MoCA increase of, or beyond, the three thresholds. The lower graphs present the data of the counterpart patients with perioperative MoCA decrease. For each column with the same threshold of increase and decrease, the combined median %CEIm and the combined median %TensI↑ were computed and were used to divide the graphs into four quarters. The quarter with both %CEIm and %TensI↑ above the median was marked in each graph, and the ratio of points in it out of the total points in the graph was compared between the increase and decrease graphs of each column, using the chi‐square test for evaluating statistical significance. For each perioperative MoCA change threshold, the ratio of patients who had above median preoperative %CEIm and postoperative %TensI↑ was significantly higher in the decrease group than in the increase group.

## 4 DISCUSSION

This study demonstrated that the electrophysiological markers CEI and TensI might be associated with performance in the MoCA neuropsychological assessment. As detailed in Section 1, the CEI marker may be associated with cognitive effort, and the TensI might be associated with alertness and might be affected by the level of stress. The first major finding of this study was that for patients in the preoperative intermediate MoCA range, higher %CEIm (cognitive effort index in the middle range), measured during the preoperative MoCA test, is associated with postoperative decrease in the MoCA score. The second major finding of this study was that increased %TensI↑ (TensI in the higher range) is associated with reduced performance in preoperative and postoperative MoCA tests.

For patients with preoperative MoCA score in the intermediate range, greater ratio of CEI in the effective middle range, during the preoperative MoCA test, was shown to be associated with perioperative MoCA decrease. If, as suggested, CEI could be associated with cognitive effort during the MoCA test, it means that patients who invest greater effort for achieving an intermediate MoCA score might be more susceptible to perioperative MoCA decrease. Alternatively, patients in this range of performance who invest less effort are less likely to demonstrate perioperative MoCA decrease. Possibly, reduced preoperative effort might lead to a misleadingly lower MoCA score and thereby might mask a postoperative cognitive decrease.

As was presented in Section 1, the literature emphasizes the role of motivation and volition on cognitive effort. There is a question of whether, and to what degree, the patient's cognitive effort represents a trait (e.g., “easy going” vs. “hard working”) or a highly variable situation‐dependent state—for example, a response to the MoCA test that may be different in other tasks, which also require cognitive effort. It is rather accepted that, at least to a large extent, cognitive effort represents a trait, or cognitive style (Lafond‐Brina et al., 2023; Yeo & Neal, 2008). In which case, the results of this study may indicate that patients of the intermediate cognitive range, whose performance depends upon cognitive effort, may be at greater risk for cognitive decrease, at least for a short duration after surgery. The dependence of the MoCA score upon cognitive effort might accord with the finding that patients at different preoperative cognitive levels showed decreases in different MoCA subscores, related with different cognitive functions. This may mean that the perioperative deterioration is not related to a highly specific, or highly localized brain dysfunction, but rather potentially to a more basic process, which may participate in multiple functions. Sustained attention might be a good candidate for such a process, as it impacts multiple other neuropsychological processes. However, as discussed in Section 1, cognitive effort seems to have a major impact upon sustained attention.

As opposed to the findings described above for the patients in the intermediate preoperative MoCA range, patients in the preoperative MoCA higher and lower ranges who demonstrate perioperative MoCA increase may tend to maintain the CEI at the effective range, more than their counterparts with perioperative MoCA decrease. These tendencies did not reach statistical significance, possibly due to smaller sample size in these groups. We could predict a priori that lower range patients who maintain cognitive effort in the effective range would be more likely to demonstrate perioperative MoCA increase. We previously described (Gvion & Shahaf, 2023—tab. 1—column 1, row 3—and related text) the situation in which effective cognitive effort co‐occurs with lacking performance, and suggested this mismatch might be because such patients allocated effort, but not to the task at hand, but possibly elsewhere. Thus, we suggested that the lacking performance does not truly represent the patient's underlying abilities. In which case, it is not surprising that the postoperative MoCA results might be better. Note that this subgroup of patients with lower range preoperative MoCA score and with perioperative MoCA increase were also the only ones to demonstrate a significant increase in attention from the preoperative test to the postoperative test, as measured by this MoCA subscore, which might suggest they may have been much more oriented to the task during the later assessment. This group of patients was also the only one to demonstrate significantly less depressive signs, in comparison with their counterparts with perioperative MoCA decrease. Hypothetically, their cognitive effort and attention before surgery may have not been allocated to the MoCA test, but rather to their general and affective condition, especially considering the fact that MoCA was administrated just after the HADS questionnaire.

We did not predict a priori the finding that patients in the higher preoperative MoCA range who demonstrated perioperative MoCA increase may also tend to have a more effective range preoperative cognitive effort than their counterparts with MoCA decrease. It might be that those patients actually attended the preoperative test better, and due to their good baseline, may have learned it in greater depth, and thereby could have obtained better results the second time they were tested, in the postoperative period. However, as stated, the results for the lower and higher preoperative ranges did not reach statistical significance, and require first establishment with a larger sample size.

Concerning the TensI results, it decreased from the preoperative cognitive assessment to the postoperative assessment in patients from the intermediate range, in whom the MoCA score increased. Furthermore, for this group of patients, higher postoperative TensI was associated with perioperative MoCA decrease. These findings may accord with the derivation of TensI as a marker for alertness, and an increased TensI may indeed provide an indication of the stress impact. It is not general anxiety or depression, as HADS scores did not demonstrate such a comprehensive association with perioperative MoCA change, but instead potentially a specific cognitive test‐related stress effect.

Notably, higher postoperative TensI was more associated with limited perioperative MoCA decrease (thresholds of at least two‐point change) and not with a very strict threshold (of at least three points). It might be that larger decreases can cause severe dysfunction and potentially some level of functional anosognosia, caused by the reduction of cognitive function.

A similar but milder and nonsignificant tendency of higher postoperative TensI in patients with perioperative MoCA decrease might also be seen in patients in the lower range of preoperative MoCA score. However, the opposite, and still nonsignificant tendency might be seen in patients in the higher range of preoperative MoCA score. It might be that, with a higher level of baseline performance, stress may not hinder the performance, or may even lead to more recruitment. However, a larger sample size is needed for assessment of these initial tendencies.

Assuming the interpretation of TensI findings as indicating of stress effect on perioperative cognitive performance would be validated with further studies, it seems to point out a “chicken and egg problem.” Is the stress a major cause of POCD, or is it caused by POCD? However, practically, it might be more useful to rephrase the problem as a “snowball problem.” That is, whatever the initial cause, stress is known to hinder performance, and may lead to further deterioration, also cognitively, as might be the case, for example, in pseudodementia, as another example. However, it might be the case that such stress‐related barriers to cognitive performance could have a rather effective treatment with focused rehabilitation sessions, which may teach the patient effective strategies to reduce the cognitive‐related stress, and thereby might “melt the snowball” (Gvion & Shahaf, 2023—tab. 1—columns 1/2, row 1—and related text and see also case report 2 therein).

All in all, the results of this study seem to indicate that it might be both advisable and feasible to monitor cognitive effort and stress‐related effects during cognitive assessment, as these two factors might have a significant effect upon the measured performance. Decades of studies suggested EEG markers might assist in assessment of specific cognitive functions (Shenal et al., 2001). Some of these markers also obtained regulatory approval for specific assistance in cognitive diagnosis, with the hope of their widespread clinical acceptance (Karceski, 2016). In this study, we undertook a somewhat different approach. We focused on potential markers, for reduced cognitive effort, and enhanced test‐related stress effect, which may hinder performance in various cognitive and behavioral assessments, regardless of their specific content. These markers do not assist in the diagnosis of a specific function, or dysfunction. Instead, they may indicate whether the assessment results truly represent the patient's best abilities, or, alternatively, whether the patient's true abilities might actually be better than the assessment results, but underestimation resulted from reduced cognitive effort, or from test‐related stress effect. In this regard, the use of perioperative MoCA could be considered as merely an example, and the approach and potential indices may have much broader application for cognitive and behavioral assessment. Specifically for POCD, diagnostic criteria and precision are under debate and constant review, and there is no clear‐cut criteria (Rudolph et al., 2010). It might be that POCD, or at least its milder form of limited decrease in cognitive score, is underdiagnosed in the elderly population. We have seen the significant age impact upon the tendency for perioperative MoCA decrease in the patients of the higher preoperative MoCA range. This may mean that elderly patients, who can demonstrate a perioperative MoCA decrease, will generally do so. Patients may not demonstrate MoCA decrease because of: (i) learning the test, due to the test–retest effect, which also occurs when using different versions of the test (Cooley et al., 2015); (ii) low cognitive effort during the preoperative assessment; and (iii) high degree of stress effect during the preoperative assessment. Thus, it is possible that many more patients suffer from undiagnosed cognitive deficits, under current anesthesia and surgery regimens, even if potentially mild and transient.

This was only an initial study, which may indicate the need for further research on monitoring cognitive effort and stress‐related impact during cognitive assessment in general, and specifically during perioperative assessments. There is certainly a need for larger prospective studies. Specifically, with regard to TensI, it might be of value to assess its association with prevailing autonomic markers for stress.

## AUTHOR CONTRIBUTIONS

**Dana Baron‐Shahaf**: Conceptualization; investigation; writing – original draft; methodology; validation; visualization; writing – review and editing; formal analysis; supervision; resources; project administration; data curation; funding acquisition. **Goded Shahaf**: Conceptualization; investigation; writing – original draft; methodology; validation; visualization; writing – review and editing; software; formal analysis; project administration; supervision; resources; data curation; funding acquisition.

## CONFLICT OF INTEREST STATEMENT

The authors are spouses. Both authors, and especially Goded Shahaf, have been involved in the past with companies, which implemented EEG‐based markers for multiple applications. However, they have not been involved with these companies over the last 4 years. The current study was performed in the Academic Research Laboratory at Rambam Healthcare Campus, and is unrelated to any of these companies.

### PEER REVIEW

The peer review history for this article is available at https://publons.com/publon/10.1002/brb3.3649.

## Data Availability

The data that support the findings of this study are available from the corresponding author upon reasonable request.
